# Evaluation of GlcNAc-Configured Glycomimetics as Pharmacological Chaperones of NAGLU for the Treatment of Mucopolysaccharidosis IIIB

**DOI:** 10.3390/biom16020313

**Published:** 2026-02-16

**Authors:** Nissrine Ballout, Jérôme Désiré, Angela Johana Espejo-Mojica, Katherin Niño-Traslaviña, Daniel Sandoval, Carlos Javier Alméciga-Díaz, Yves Blériot, Jérôme Ausseil

**Affiliations:** 1 Toulouse Institute for Infectious and Inflammatory Diseases (Infinity), University of Toulouse, Inserm U1291, CNRS U5051, 31024 Toulouse, France; 2 Biochemistry Laboratory, Federative Institute of Biology, CHU Toulouse, 31024 Toulouse, France; 3 Organic Synthesis Team, Glycochemistry Group, IC2MP UMR CNRS 7285, Université of Poitiers, 86073 Poitiers Cedex 9, France; 4 Institute for the Study of Inborn Errors of Metabolism, Faculty of Science Pontificia Universidad Javeriana Cra. 7 No. 43-82 Building 54, Lab 305A, Bogotá 110231, Colombiacjalmeciga@javeriana.edu.co (C.J.A.-D.); 5 Dogma Biotech, Bogotá 110231, Colombia

**Keywords:** mucopolysaccharidosis IIIB, *N*-acetyl-glucosaminidase (NAGLU), pharmacological chaperone therapy (PCT), glycomimetics

## Abstract

The interaction of a set of four *N*-acetyl-glucosamine (GlcNAc) glycomimetics with human *N*-acetyl-glucosaminidase (NAGLU), the genetically defective enzyme in patients suffering from mucopolysaccharidosis (MPS) IIIB, also known as Sanfilippo B syndrome, was investigated to identify potential pharmacological chaperones. Glycomimetic–NAGLU binding was initially studied by molecular docking simulations and a thermal shift assay. The effects of the glycomimetics on NAGLU activity enhancement were studied in fibroblast cells from seven MPS IIIB patients. A significant increase in NAGLU activity in four cell lines in the presence of glycomimetic MK 8719, a molecule tested in a Phase 1 study in healthy volunteers to treat Alzheimer’s disease, was demonstrated. Furthermore, MK 8719 prevented the increase in glycosaminoglycan (GAG) levels in four MPS IIIB fibroblast cells, suggesting that this molecule may be worth investigating further as a pharmacological chaperone for MPS IIIB. These results represent an important contribution towards the development of a specific therapy for MPS IIIB.

## 1. Introduction

Lysosomal storage disorders (LSDs) are a group of 70 inherited monogenic disorders. Each is caused by a deficiency in a lysosomal enzyme responsible for the degradation of a metabolic substrate, leading to the accumulation of this substrate and subsequent lysosomal malfunction and disease [1,2]. Mucopolysaccharidosis type III (MPS III), or Sanfilippo syndrome, includes a group of four autosomal recessive neurodegenerative LSDs whose symptoms are due to the incomplete lysosomal degradation of heparan sulfate (HS) [3]. HS is a complex glycosaminoglycan (GAG) involved in several mechanisms such as repair biology, inflammation, tissue remodeling, and cellular development and growth [4]. Mucopolysaccharidosis type IIIB (MPS IIIB, Sanfilippo syndrome type B) is caused by mutations in *N*-acetyl-alpha-glucosaminidase (NAGLU), which breaks down HS. HS accumulation has been linked to several central nervous system (CNS) manifestations [5], and its malfunctioning results in a progressive impairment of the CNS, causing a progressive effect on the rest of the body’s systems. The resulting neurological symptoms of MPS IIIB highlight the need for a CNS-directed therapeutic solution [6]. MPS IIIB is genetically heterogeneous, with 229 disease-associated variants identified; most of the known mutant alleles in MPS IIIB patients occur at low frequencies or not more than once [7].

The estimated incidence rate for MPS IIIB is 1 in 200,000 newborns [8]. This devastating disease concerns about 1000–2000 patients in the developed world, who generally only survive into early adulthood. Clinical manifestations of MPS IIIB affect multiple organ systems, including the cardiovascular and respiratory systems [9]. They can be divided into three distinct phases [10]. In phase I, between ages two and six, the symptoms can include hearing loss, speech and language deficits, and motor deficits. Phase II onset begins between the ages of eight and the early teens, and is characterized by the regression of or failure to reach developmental milestones, pronounced periods of hyperactivity and aggressive behavior, and the progression of organomegaly. The final phase of MPS IIIB is marked by severe dementia, loss of motor function, and seizure activity. Death is due to the severe neurological degeneration resulting from the build-up of HS, but is often proximately caused by respiratory tract infections, such as pneumonia, resulting from diminished airway protection.

There is currently no specific treatment approved for MPS IIIB, and early intervention is necessary to prevent irreversible neuronal damage and neuroinflammation caused by the progressive build-up of HS [11]. However, several treatment approaches for MPS IIIB have been pursued, including hematopoietic stem cell transplants (HSCTs), enzyme replacement therapy (ERT), substrate reduction therapy (SRT), gene therapy (GT), and pharmacological chaperone therapy (PCT) [12,13]. HSCTs have successfully aided in the treatment of several LSDs, but have been disappointing for the treatment of MPS III, failing to reverse neurological dysfunction or prevent additional neurocognitive decline when administered later in life [14]. In MPS IIIB specifically, HSCTs have not demonstrated efficacy, largely due to poor CNS correction, diagnostic delays, and a high procedural risk, and are therefore not currently considered a standard therapy [15]. Another option for treating MPS IIIB is enzyme replacement therapy (ERT). A phase 1/2 open-label clinical study showed that intravenous administration of recombinant NAGLU reduced visceral HS storage; however, neurocognitive improvements were limited, and the reduction in the HS levels in the cerebrospinal fluid was both transient and negligible [16]. ERT remains the most widely implemented strategy, providing clinical benefit by supplying recombinant enzymes. Its efficacy, however, is limited by its poor penetration across the BBB (particularly important for neuronopathic LSDs), immunogenic responses, and high cost. To better address CNS involvement, modified delivery approaches and engineered enzymes have been developed (BMN 250), though these remain invasive and not broadly applicable [11,17]. SRT offers an oral alternative by reducing the synthesis of accumulating metabolites, although its efficacy is incomplete, requiring residual activity, and its off-target effects remain problematic. Specifically, genistein supplementation, a strategy shown to inhibit glycosaminoglycan synthesis in patient fibroblasts, was evaluated in clinical trials, but failed to demonstrate improvement in patient disability [18]. GT, particularly with adeno-associated virus (AAV)-mediated vectors, holds promise for durable enzyme replacement, but requires early intervention to preserve neurological function, which limits its applicability to all patients, particularly those diagnosed at later stages [19,20]. Finally, PCT represents a promising therapeutic strategy by stabilizing misfolded enzymes, improving lysosomal trafficking, and crossing into the brain, making this strategy particularly relevant for patients carrying residual, rescuable enzyme activity [21,22]. PCT can be considered either in monotherapy for rescuing mutated endogenous enzyme functional states or in dual therapy together with recombinant enzymes to enhance its bioavailability [22,23].

Of the 230 pathogenic variants identified in MPS IIIB, around 169 are missense mutations [7]. These are clinically significant because some lead to complete catalytic loss, whereas others primarily impair folding or stability while preserving residual activity. The latter group represents the most promising targets for PCT. Importantly, even modest restoration of enzyme activity (less than 20% of normal) is predicted to prevent substrate accumulation, as shown in other LSDs [24]. PCT has been used in MPS IIIC patient fibroblasts with promising results that encourage further research into the topic [25]. In addition, PCT has shown clinical benefit in the Fabry, Gaucher, and Pompe diseases [26,27,28,29]. Galafold (migalastat) is an approved chaperone molecule treatment for use in patients with Fabry disease who have migalastat-amenable GLA mutations [29,30,31,32]. However, in MPSIIIB, a high-throughput screening of over 1300 compounds failed to identify effective stabilizers for NAGLU [33].

In this context, iminosugars and *N*-acetyl-glucosamine (GlcNAc) glycomimetics have attracted significant attention as candidate pharmacological chaperones for LSDs. These small molecules can mimic natural sugar substrates or intermediates, bind to amino acid residues resembling the active site of mutant enzymes, and stabilize them during folding in the endoplasmic reticulum [26]. Early experimental work with iminosugars highlighted their chaperone potential, and subsequent studies proposed both active-site and allosteric binding modes [34,35,36]. Isoiminosugars such as isofagomine have shown efficacy in stabilizing misfolded enzymes in other LSDs, including the Gaucher [37] and Pompe [38] diseases, highlighting the generalizable potential of this strategy. Notably, 4-*epi*-isofagomine exhibited strong activity in a range of β-galactosidase-compromised human cell lines and may represent a promising lead for the development of new pharmacological chaperones for GM1-gangliosidosis and Morquio B disease [39,40].

**Figure 1:**
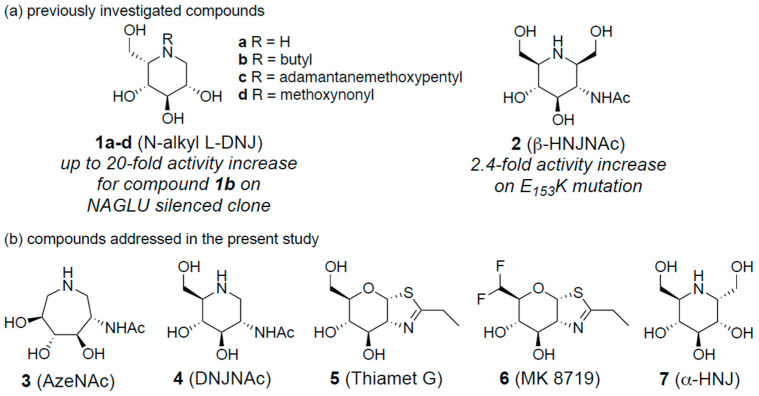
Structure of GlcNAc glycomimetics (**a**) previously studied **1**–**2** and (**b**) under investigation in this study **3**–**7**.

Our group and colleagues explored chaperone therapy in MPS IIIB by designing or identifying NAGLU inhibitors based on an iminosugar scaffold. In particular, *N*-substituted l-iminosugars **1** (Figure 1a), were found to significantly reduce substrate storage and lysosomal dysfunctions in MPS III fibroblasts and a neuronal cellular model of the MPS IIIB subtype. In addition, these glycomimetics increased the levels of defective *α*-*N*-acetylglucosaminidase, corrected its proper sorting toward the lysosomal compartment, and reduced HS accumulation by downregulating protein levels of exostosin glycosyltransferases [36]. From our side, capitalizing on the mechanism of NAGLU and the three-dimensional structure of its postulated transition state [34], we designed and synthesized homoiminosugar-based NAGLU inhibitors **2** (Figure 1a), that proved to promote a two-fold activity enhancement of mutant NAGLU at their optimal concentration [41]. However, the synthetic efforts, including the number of synthetic steps to access these derivatives and analogs, are hardly compatible with further drug development. In addition, the limited number of pathogenic variants that were tested in our initial study does not match the genetic heterogeneity found in MPS IIIB. Altogether, these features led us to design a new study focused on simpler GlcNAc glycomimetics, either readily accessible or commercially available, to be tested across a broader panel of patient-derived pathogenic variants.

We selected four glycomimetics **3**–**6** that are known to act as GlcNAc mimics, namely in-house, easily available azepane AzeNAc **3** [42], and commercial compounds: 2-acetamido-1,2-dideoxynojirimycin (DNJNAc) **4** [43], Thiamet-G **5** [44], and MK 8719 **6** [45]. AzeNAc **3** is a potent inhibitor of several hexosaminidases [46], as is DNJNAc **4** [43]. Both Thiamet-G **5** and MK 8719 **6** have been designed to inhibit *O*-linked *N*-acetylglucosaminidase (*O*-GlcNAcase; OGA), a glycosidase involved in tauopathies. Importantly, MK 8719 is an optimized derivative of Thiamet G, developed through a strategy aimed at reducing its polar surface area to enhance key drug-like properties, including the potency, the selectivity, a high CNS exposure, metabolic stability, favorable pharmacokinetics, and a strong in vivo pharmacodynamic response [47]. MK 8719 is a potent inhibitor of the human OGA enzyme with comparable activity against the corresponding enzymes from mice, rats, and dogs. In vivo studies showed that oral administration of MK 8719 elevates brain and peripheral blood mononuclear cell *O*-GlcNAc levels in a dose-dependent manner. In addition, positron emission tomography imaging studies demonstrated robust target engagement of MK 8719 in the brains of rats. Thiamet G **5** and MK 8719 **6** [47] do not significantly inhibit NAGLU at a 1 mM concentration, while AzeNAc **3** and DNJNAc **4** are micromolar inhibitors of this enzyme [41]. The naturally occurring α-homonojirimycin **7**, which is deprived of the NHAc group and not an inhibitor of GlcNAc processing enzymes, was used as a negative control (Figure 1b) [48].

## 2. Materials and Methods

### 2.1. Molecular Docking

Molecular docking was performed as previously reported [49,50,51]. Briefly, the NAGLU structure was retrieved from the Protein Data Bank (PDB 4XWH) [52]. Compound structures were retrieved from PubChem: AzeNAc (**3**, PubChem CID 42632559), DNJNAc (**4**, PubChem CID 122617), Thiamet G (**5**, PubChem CID 135566354), MK 8719 (**6**, PubChem CID 136416849), and α-homonojirimycin (α-HNJ–**7**, PubChem CID 159496). Molecular docking was performed using Autodock vina, v1.1.2 [53], with a gridbox set on the enzyme’s active site. The results of the NAGLU–compound interaction are reported as the affinity energy (kcal/mol) and were analyzed by using Maestro, version 13.5.128 (Schrödinger, New York, NY, USA). The most probable conformation of each docking was selected by comparing against the molecular docking of HS, performed as previously reported [51].

### 2.2. Thermal Shift Assay

The compounds were tested in a thermal shift assay to evaluate their binding to a recombinant human NAGLU (hrNAGLU). The thermal shift assay was performed as previously described [51], by using an hrNAGLU produced in the yeast *Komatagaella phaffii* following previously reported protocols [54,55]. The hrNAGLU in 25 mM citrate buffer, with a pH of 5.5, was mixed with Sypro Orange 200X (Molecular Probes, ThermoFisher Scientific, Eugene, OR, USA) and the selected compounds at 0.1, 1.0, and 10 mM concentrations. Every concentration was done in triplicate. hrNAGLU without any compound was used as the negative control. The mixtures were incubated from 20 to 90 °C at 1°/min using a QuantStudio™ 3 Real-Time PCR system (Applied Biosystems, ThermoFisher Scientific, Waltham, MA, USA). Melting temperatures (T_m_) were calculated by using QuantStudio Design & Analysis available at Thermo Fisher Connect (ThermoFisher Scientific, Waltham, MA, USA).

### 2.3. Cell Culture

For screening assays, eight fibroblast cell lines were used: one from a healthy donor (used as a positive control) and seven from MPS IIIB patients carrying distinct pathogenic variants in the NAGLU gene, representing a broad clinical spectrum of MPS IIIB syndrome (Table 1). Human fibroblast cells were cultured in 175 cm^2^ flasks containing 35 mL of Dulbecco’s modified Eagle medium (61965-025, ThermoFisher Scientific, Waltham, MA, USA) supplemented with 10% inactivated fetal calf serum (SVF, F7524, Merck, Darmstadt, Germany). The cells were maintained in an incubator at 37 °C, with 5% CO_2_ and 90% humidity. The medium was changed every 48 h. For the NAGLU enzyme activity assay, cells were plated at 150,000 cells per well in 6-well plates and exposed to test compounds 24 h post-seeding. The treatment was performed at three concentrations (10, 30, and 100 µM), and the effects were assessed at two time points: 48 and 72 h after exposure. All the experiments were conducted in four independent replicates. GAG accumulation was evaluated following treatment with 100 µM of the chaperone compound in 25 cm^2^ flasks seeded with 5 x 10^5^ cells. The treatment was stopped 72 h post-exposure. All experiments were performed in three independent replicates.

### 2.4. NAGLU Enzyme Activity Assay

NAGLU activity in fibroblasts was measured using the fluorogenic substrate 4-methylumbelliferyl 2-acetamido-2-deoxy-α-D-glucopyranoside (4MU-α-GlcNAc, EM182013, Biosynth, Compton, UK). Cell lysis was performed by adding 150 µL of ultrapure water to each well, followed by scraping and three freeze–thaw cycles by alternating between immersion in liquid nitrogen and thawing at 37 °C. The lysates were centrifuged at 13,000× *g* for 3 min and the supernatants were collected for analysis. The protein concentration was determined using the Pierce™ Rapid Gold BCA Protein Assay Kit (A53226, ThermoFisher Scientific, Waltham, MA, USA) according to the manufacturer’s instructions. For the enzyme activity assay, 50 µL of each sample was mixed with 50 µL of 0.2 mM 4MU-α-GlcNAc substrate in 0.2 M sodium acetate buffer (pH of 4.3). The reactions were incubated at 37 °C for 17 h in the dark. After stopping the reaction by adding 200 µL of 1 M glycine buffer (pH of 10.0), the released fluorescence was measured with an excitation wavelength of 355 nm and emission at 460 nm using a Varioskan (VLB000D1, ThermoFisher Scientific, Waltham, MA, USA). The NAGLU activity in each sample was calculated using a calibration curve of 4-methylumbelliferone (M1381, Merck, Darmstadt, Germany). All the enzyme activities were normalized against the total protein content.

### 2.5. Glycosaminoglycan (GAG) Accumulation

The total GAGs were quantified in human fibroblasts using a 1,9-dimethylmethylene blue (DMB) dye-binding assay. After delipidation and deproteinization, the cell pellets were air-dried for at least 4 h. The dried samples were then digested in 1 mL of papain digestion buffer (0.1 M sodium acetate (S8625, Merck, Darmstadt, Germany), 5 mM EDTA (E6635, Merck, Darmstadt, Germany), 5 mM cysteine (168149, Merck, Darmstadt, Germany), and 0.1 mg/mL papain (P3375, Merck, Darmstadt, Germany), pH of 5.5) and incubated at 65 °C for 24 h to solubilize intracellular GAGs. After digestion, the samples were centrifuged, and the supernatants were mixed with 200 µL of DMB reagent (20335.01, Serva) in formate buffer, with a pH of 3.3. The absorbance was read immediately at 525 nm using a Varioskan (VLB000D1, ThermoFisher Scientific, Waltham, MA, USA). A standard curve was prepared from serial dilutions of heparan sulfate (H7640, Merck, Darmstadt, Germany) in 0.05 M sodium acetate buffer (pH of 4.5). The GAG levels in the samples were interpolated from the standard curve and normalized to the protein content measured in parallel using the Pierce™ BCA Protein Assay Kit (A53226, ThermoFisher Scientific, Waltham, MA, USA).

### 2.6. Statistical Analysis

Data for the quantitative variables are expressed as the mean ± SEM. All the statistical analyses were performed using the GraphPad Prism software (version 10.4.1, GraphPad Software, Boston, MA, USA). Statistical differences between the groups were measured using a one-way analysis of variance (ANOVA) followed by the Kruskal–Wallis test. The threshold for statistical significance was set at *p* < 0.05.

## 3. Results and Discussion

### 3.1. Molecular Docking and Thermal Shift Assay

**Figure 2:**
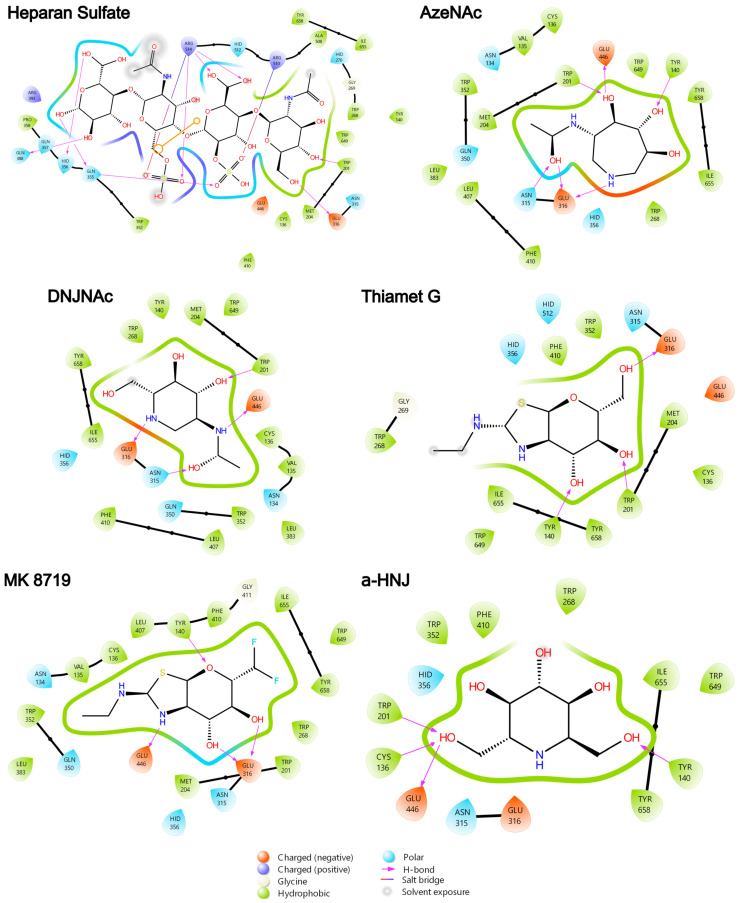
Molecular docking and prediction of the interactions of NAGLU (PDB 4XWH) with heparan sulfate (yellow) and the glycomimetics AzeNAc (**3**), DNJNAc (**4**), Thiamet G (**5**), MK 8719 (**6**), and α-homonojirimycin (α-HNJ, **7**). Catalytic residues Glu316 and Glu446, within the NAGLU active cavity, are colored in red and blue, respectively.

To model the interactions of glycomimetics **3**–**7** with human NAGLU, we performed a molecular docking simulation based on previous studies for NAGLU [51] and GALNS (MPS IVA) [49] pharmacological chaperone studies. The simulations showed that all the glycomimetics docked at the bottom of the active site, similarly to the GlcNAc motif of HS and next to the catalytic residues Glu316 and Glu446 (Figure S1). As previously reported [51], the analysis of the interactions predicted that GlcNAc from HS mainly interacts through hydrophobic interactions with Cys136, Tyr140, Trp201, Met204, Trp268, Phe410, Ala508, Trp649, Ile655, and Tyr658. Additionally, polar (His270 and Asn315) and charged (Glu316 and Glu446) amino acids were involved (Figure 2). Most of these interactions were also predicted with the glycomimetics **3**–**7**, highlighting the interactions with Cys136, Tyr140, Trp268, Asn315, Glu316, Phe410, Ile655, and Tyr658, which were observed in all the compounds (Figure 2). Trp352 and His356, which were predicted to interact with non-GlcNAc motifs from HS, also interacted with all the glycomimetics. The highest affinity was predicted for HS (*K*_i_ = 0.93 µM), followed by DNJNAc (**3**, *K*_i_ = 9.94 µM), AzeNAc (**4**, *K*_i_ = 23.2 µM), MK 8719 (**5**, *K*_i_ = 32.5 µM) and Thiamet G (**6**, *K*_i_ = 45.7 µM). Noteworthy, α-HNJ **7**, which was used as a negative control since it lacks the NHAc group and is not an inhibitor of GlcNAc processing enzymes, showed the lowest affinity with a *K*_i_ of 64.1 µM that was between 1.5- and 2.8-fold higher than that predicted for glycomimetics **3**–**6**, and about 70-fold higher than that of HS. An interesting observation was a change in the H-bond profile. Whereas the GlcNAc motif at HS only interacts with Glu316 through an H-bond, three to four H-bonds were predicted for glycomimetics **3**–**7** involving Tyr140/Trp201, Asn315, Glu316, and Glu446. Overall, these results showed that glycomimetics **3**–**7** may bind to human NAGLU by performing interactions like those predicted for HS, but with different affinities.

**Figure 3:**
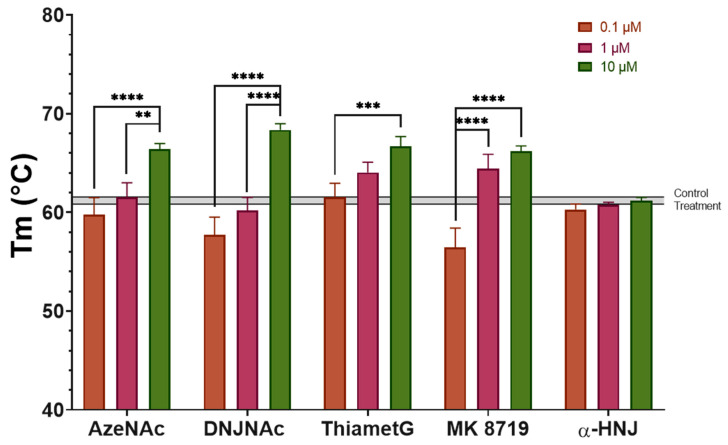
Thermal shift assay. The melting temperature (T_m_) of the hrNAGLU enzyme was measured in response to different concentrations of the glycomimetics AzeNAc (**3**), DNJNAc (**4**), Thiamet G (**5**), MK 8719 (**6**), and α-homonojirimycin (α-HNJ, **7**). Untreated hrNAGLU was used as the negative control (T_m_ = 61.2 ± 0.4 °C), represented by the grey line. Significant differences correspond to the one-way ANOVA test. ** *p* < 0.01, *** *p* < 0.001, **** *p* < 0.0001.

The ability of glycomimetics **3**–**7** to protect rhNAGLU against temperature-induced denaturation was evaluated in vitro, through a thermal shift assay using Sypro Orange dye. The results showed that incubation with 0.1 and 1 µM of the glycomimetics did not induce a significant increase in the T_m_ of the hrNAGLU, except for Thiamet G (**5**) and MK 8719 (**6**), which led to a 2.8 ± 1.0 (*p* = 0.0137) and 3.3 ± 1.4 (*p* = 0.0041) °C increase in T_m_ compared to the control treatment (T_m_ = 61.2 ± 0.4 °C) at a 1 µM concentration. On the other hand, treatment with 10 µM of glycomimetics **3**–**6** significantly increased (*p* < 0.0001) the T_m_ of the hrNAGLU by 5.2 ± 0.6, 7.2 ± 0.6, 5.5 ± 1.0, and 5.0 ± 0.5 °C for AzeNAc (**3**), DNJNAc (**4**), Thiamet G (**5**), and MK 8719 (**6**), respectively, compared to the control experiment (Figure 3). Although molecular docking suggested that α-HNJ (**7**) may bind to the active cavity of NAGLU, the thermal shift assay results showed that, under the evaluated conditions (up to 10 μM) this compound does not bind to rhNAGLU, which agrees with the lower binding affinity predicted and the lack of inhibition activity of GlcNAc processing enzymes. Overall, these results suggest that glycomimetics **3**–**6** are direct binders of NAGLU and may function as PCs.

Molecular docking simulations and the thermal shift assay showed that the evaluated glycomimetics may work as pharmacological chaperones of NAGLU, since these compounds may perform similar interactions to those predicted for HS and induce significant Tm changes. Previously, the identification of atovaquone and piperaquine as potential pharmacological chaperones of NAGLU was reported [51]. Atovaquone and piperaquine induced a 7.1 and 8.8 °C Tm increase by using the same hrNAGLU used in the present study. Noteworthy, these Tm increases were similar to those observed for the glycomimetics described in the present study, which were between 2.8 and 7.2 °C. Similar Tm increases were also reported for pharmacological chaperones described for Fabry [56] and MPS IV A [49]. However, higher increases in NAGLU activity were observed after treatment with glycomimetics compared to the increase observed with atovaquone and piperaquine [51]. These results suggest that, although both glycomimetics and atovaquone and piperaquine interact with NAGLU, increasing the enzyme thermal stability, there are other factors involved in the restauration of the enzyme activity. These additional factors may be associated with the chaperone–protein interactions. The molecular docking simulations showed that the evaluated glycomimetics generated specific H-bond interactions that not only differed from those predicted for HS, but also were not predicted for atovaquone and piperaquine [51]. In this sense, further studies may focus on the study of the impact of H-bonds on the chaperone activity of these glycomimetics.

### 3.2. NAGLU Activity Enhancement in MPS IIIB Patient Cells

To rule out any cytotoxic effects of the chaperone molecules on fibroblast cells, a cell proliferation assay (water-soluble tetrazolium salt 1, WST-1) was performed. In brief, cells were treated with either the culture medium (NT) or the tested molecule at 10, 30, or 100 µM for 48 and 72 h (h). No significant differences were observed between the different treatment conditions and the incubation times (Figure S2).

Next, we investigated the NAGLU enzyme activity after treatment of MPS IIIB fibroblast cells with five different compounds.

#### 3.2.1. Compound **3** (AzeNAc)

**Figure 4:**
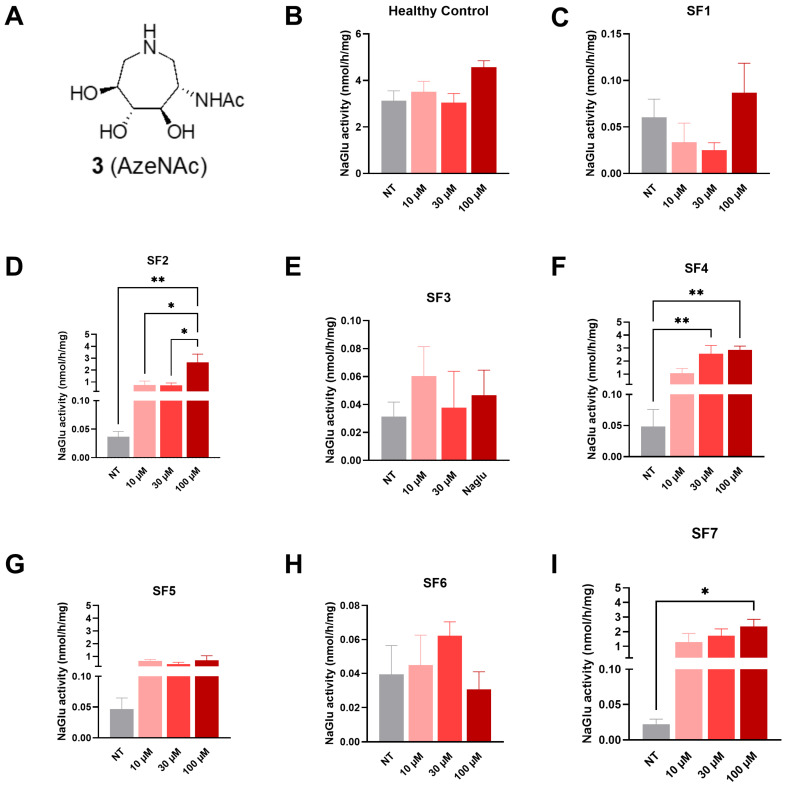
NAGLU enzyme activity measured 72 h after treatment with compound **3** (AzeNAc), tested at three concentrations: 10, 30, and 100 µM. (**A**) AzeNAc structure, (**B**) healthy control fibroblasts, (**C**) SF1 fibroblasts, (**D**) SF2 fibroblasts, (**E**) SF3 fibroblasts, (**F**) SF4 fibroblasts, (**G**) SF5 fibroblasts, (**H**) SF6 fibroblasts, and (**I**) SF7 fibroblasts. Experiments were conducted in four independent replicates. Data are expressed as the mean ± SEM (*n* = 4). The *p*-value was determined using a one-way ANOVA, followed by Tukey’s test for multiple comparisons (* *p* < 0.05, ** *p* < 0.01). NT = non-treated.

At 72 h post-treatment, a significant increase in NAGLU enzyme activity was detected in SF2 fibroblasts at 100 µM (2.64 nmol/h/mg, Figure 4D), in SF4 fibroblasts at both 30 (2.56 nmol/h/mg) and 100 µM (2.85 nmol/h/mg, Figure 4F), and in SF7 fibroblasts at 100 µM (2.35 nmol/h/mg, Figure 4I) in comparison to non-treated cells (0.02–0.04 nmol/h/mg).

#### 3.2.2. Compound **4** (DNJNAc)

**Figure 5:**
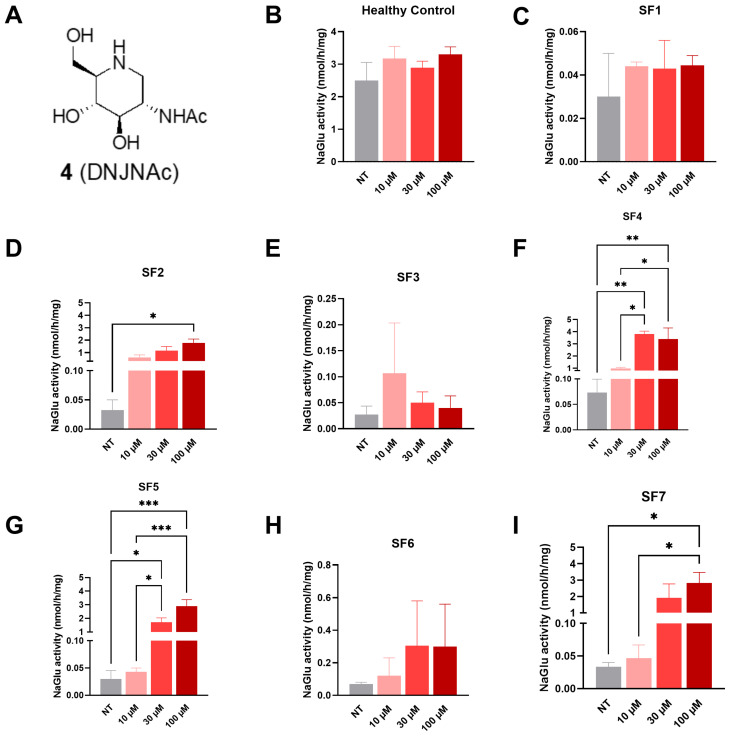
NAGLU enzyme activity measured 72 h after treatment with compound **4** (DNJNAc) tested at three concentrations: 10, 30, and 100 µM. (**A**) DNJNAc structure, (**B**) healthy control fibroblasts, (**C**) SF1 fibroblasts, (**D**) SF2 fibroblasts, (**E**) SF3 fibroblasts, (**F**) SF4 fibroblasts, (**G**) SF5 fibroblasts, (**H**) SF6 fibroblasts, and (**I**) SF7 fibroblasts. Experiments were conducted in four independent replicates. Data are expressed as the mean ± SEM (*n* = 4). The *p*-value was determined using a one-way ANOVA, followed by Tukey’s test for multiple comparisons (* *p* < 0.05, ** *p* < 0.01, *** *p* < 0.001). NT = non-treated.

At 72 h post-treatment, a significant increase in NAGLU enzyme activity was detected in SF2 fibroblasts at 100 µM (1.78 nmol/h/mg, Figure 5D), in SF4 fibroblasts at both 30 (3.81 nmol/h/mg) and 100 µM (3.39 nmol/h/mg, Figure 5F), in SF5 fibroblasts at both 30 (1.72 nmol/h/mg) and 100 µM (2.89 nmol/h/mg, Figure 5G), and in SF7 fibroblasts at 100 µM (2.82 nmol/h/mg, Figure 5I) in comparison to non-treated cells (0.02–0.04 nmol/h/mg).

#### 3.2.3. Compound **5** (Thiamet G)

**Figure 6:**
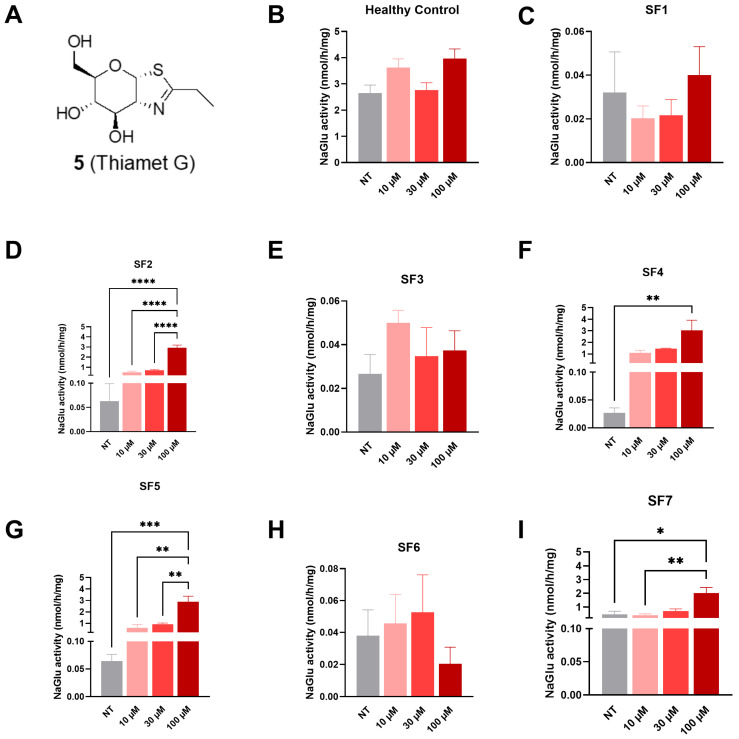
NAGLU enzyme activity measured 72 h after treatment with compound **5** (Thiamet G) tested at three concentrations: 10, 30, and 100 µM. (**A**) Thiamet G structure, (**B**) healthy control fibroblasts, (**C**) SF1 fibroblasts, (**D**) SF2 fibroblasts, (**E**) SF3 fibroblasts, (**F**) SF4 fibroblasts, (**G**) SF5 fibroblasts, (**H**) SF6 fibroblasts, and (**I**) SF7 fibroblasts. Experiments were conducted in four independent replicates. Data are expressed as the mean ± SEM (*n* = 4). The *p*-value was determined using a one-way ANOVA, followed by Tukey’s test for multiple comparisons (* *p* < 0.05, ** *p* < 0.01, *** *p* < 0.001, **** *p* < 0.0001). NT = non-treated.

At 72 h post-treatment, a significant increase in NAGLU enzyme activity was detected in SF2 fibroblasts at 100 µM (2.93 nmol/h/mg, Figure 6D), in SF4 fibroblasts at 100 µM (3.04 nmol/h/mg, Figure 6F), in SF5 fibroblasts at 100 µM (2.87 nmol/h/mg, Figure 6G), and in SF7 fibroblasts at 100 µM (2.01 nmol/h/mg, Figure 6I) in comparison to non-treated cells (0.02–0.04 nmol/h/mg).

#### 3.2.4. Compound **6** (MK 8719)

**Figure 7:**
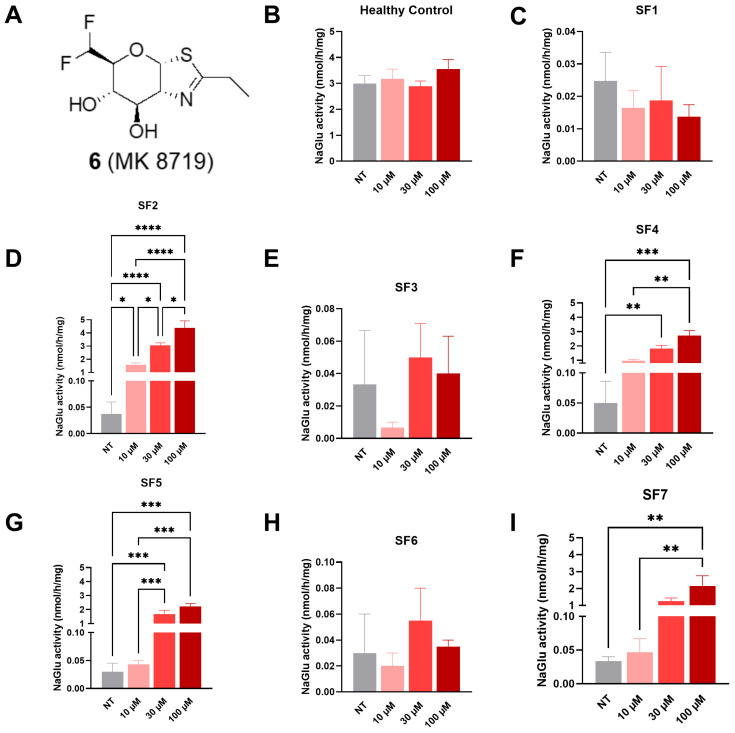
NAGLU enzyme activity measured 72 h after treatment with compound **6** (MK 8719) tested at three concentrations: 10, 30, and 100 µM. (**A**) MK 8719 structure, (**B**) healthy control fibroblasts, (**C**) SF1 fibroblasts, (**D**) SF2 fibroblasts, (**E**) SF3 fibroblasts, (**F**) SF4 fibroblasts, (**G**) SF5 fibroblasts, (**H**) SF6 fibroblasts, and (**I**) SF7 fibroblasts. Experiments were conducted in four independent replicates. Data are expressed as the mean ± SEM (*n* = 4). The *p*-value was determined using a one-way ANOVA, followed by Tukey’s test for multiple comparisons (* *p* < 0.05, ** *p* < 0.01, *** *p* < 0.001, **** *p* < 0.0001). NT = non-treated.

At 72 h post-treatment, a significant increase in NAGLU enzyme activity was detected in SF2 fibroblasts starting at 10 µM (1.58 nmol/h/mg) and at both 30 µM (3.05 nmol/h/mg) and 100 µM (4.39 nmol/h/mg, Figure 7D), in SF4 fibroblasts at both 30 (1.81 nmol/h/mg) and 100 µM (2.72 nmol/h/mg, Figure 7F), in SF5 fibroblasts at both 30 (1.66 nmol/h/mg) and 100 µM (2.22 nmol/h/mg, Figure 7G), and in SF7 fibroblasts at 100 µM (2.15 nmol/h/mg, Figure 7I) in comparison to non-treated cells (0.02–0.04 nmol/h/mg).

#### 3.2.5. Compound **7** (α-HNJ)

**Figure 8:**
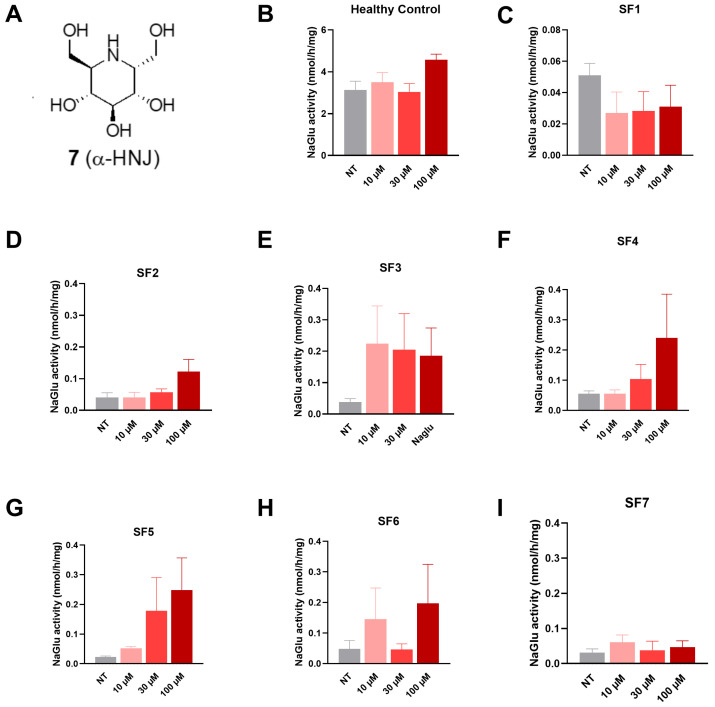
NAGLU enzyme activity measured 72 h after treatment with compound **7** (α-HNJ) tested at three concentrations: 10, 30, and 100 µM. (**A**) α-HNJ structure, (**B**) healthy control fibroblasts, (**C**) SF1 fibroblasts, (**D**) SF2 fibroblasts, (**E**) SF3 fibroblasts, (**F**) SF4 fibroblasts, (**G**) SF5 fibroblasts, (**H**) SF6 fibroblasts, and (**I**) SF7 fibroblasts. Experiments were conducted in four independent replicates. Data are expressed as the mean ± SEM (*n* = 4). The *p*-value was determined using a one-way ANOVA, followed by Tukey’s test for multiple comparisons. NT = non-treated.

This compound, which lacks the NHAc group and does not inhibit GlcNAc processing enzymes, was used as a negative control. No significant increase in NAGLU enzyme activity was detected in any MPS IIIB fibroblast line at 72 h post-treatment (Figure 8).

Herein, we demonstrated the efficacy of four glycomimetics that are known to act as GlcNAc mimics on four out of seven different MPS IIIB patient cells by showing restoration of NAGLU enzyme activity (Table 2). Our data show a clear genotype–phenotype relationship for pharmacological chaperone responsiveness in MPS IIIB fibroblasts. Lines harboring truncating or splice defects (SF1, SF3, and SF6) were uniformly non-responsive to all the tested compounds, consistent with the absence of a full-length, foldable NAGLU polypeptide. In contrast, cell lines bearing missense substitutions (SF2, SF4, SF5, and SF7) showed varying degrees of rescue by one or more compounds. Three compounds (DNJNAc, Thiamet G, and MK 8719) displayed the broadest activity across rescuable genotypes. The results of the enzyme activity measurements revealed that DNJNAc in the SF4 cell line resulted in a 52-fold increase in enzyme activity compared to untreated cells at a concentration of 100 μM. Regarding Thiamet G, in the SF4 cell line, we observed a 113-fold increase in enzyme activity compared to untreated cells at a concentration of 100 μM. Finally, MK 8719 demonstrated the highest increase in activity at a concentration of 100 μM in the SF2 cell line. This concentration corresponded to a 117-fold increase compared to untreated cells. Importantly, this pathogenic variant (SF2: R482W) belongs to a hotspot of clinically relevant mutations expected to disrupt the overall structural integrity of NAGLU [52]. Interestingly, the results obtained with Thiamet G and MK 8719 approached NAGLU activity levels comparable to those observed in healthy fibroblasts (Figure 6B and Figure 7B).

### 3.3. Normalization of GAG Concentration in MPS IIIB Patient Cells

**Figure 9:**
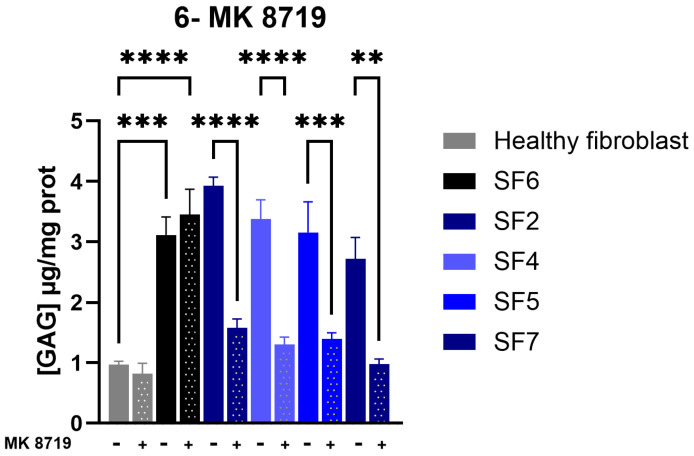
GAG accumulation levels in control (healthy fibroblast) and MPS IIIB fibroblasts not-treated (-MK 8719) or treated (+MK 8719) with MK 8719 at 100 µM for 72 h. Experiments were conducted in three independent replicates. Data are expressed as the mean ± SEM (*n* = 3). The *p*-value was determined using a one-way ANOVA, followed by Tukey’s test for multiple comparisons (** *p* < 0.01; *** *p* < 0.001; **** *p* < 0.0001).

The level of GAGs was remarkably higher in the MPS IIIB non-treated fibroblasts (SF6) compared to healthy fibroblasts (Figure 9). We then examined whether the tested compounds prevented GAG accumulation in MPS IIIB fibroblast cells following treatment. Considering the NAGLU enzyme activity data, the most effective compound, MK 8719, was selected for further experimental evaluation.

Interestingly, treatment with MK 8719 at 100 μM for 72 h prevented the accumulation of GAGs in four MPS IIIB fibroblast cell lines compared with untreated MPS IIIB fibroblast cells. However, no reduction was observed in SF6 fibroblast cells, which were used as a negative control (Figure 9). These results suggest that MK 8719 exhibits potential chaperone activity, as evidenced by increased NAGLU enzyme activity accompanied by normalization of the GAG concentration in four different MPS IIIB fibroblast lines (SF2, SF4, SF5, and SF7).

MK 8719 is a potent, selective inhibitor of *O*-GlcNAcase (OGA) developed as a CNS-penetrant small molecule to increase protein *O*-GlcNAcylation and thereby reduce pathological tau phosphorylation/aggregation in tauopathy models [45]. In the rTg4510 mouse model, MK 8719 elevates brain *O*-GlcNAc levels and reduces pathological tau and neurodegeneration [47]. While docking experiments have demonstrated that MK 8719 binds to the active site of NAGLU, this molecule has been proven to be a poor inhibitor of this enzyme [45]. Therefore, the chaperone effect produced by MK 8719 suggests that this molecule engages with an allosteric stabilizing site on NAGLU, independent of OGA inhibition.

From a translational perspective, these results support a promising approach for MPS IIIB, where patients are eligible for PCT, and their genotypes could guide the selection of these molecules. Although optimization of the compound potency, bioavailability, and CNS penetration remains necessary, glycomimetic chaperones represent a promising therapeutic direction. Notably, MK 8719, a compound with demonstrated CNS exposure in clinical studies for tauopathies, offers an encouraging precedent for safety and brain penetrance. Its ability to reach the CNS makes it especially relevant for MPS IIIB, where neurological involvement is a major therapeutic challenge. Therefore, MK 8719 may represent a novel, brain-penetrant approach capable of stabilizing specific NAGLU missense variants and potentially mitigating the neurodegenerative progression of MPS IIIB.

To better assess therapeutic relevance, MK 8719 should therefore be evaluated in neurons derived from MPS IIIB patient iPSCs to cover neuronal-specific differences in proteostasis, trafficking, and compound uptake. Furthermore, advancing chaperone therapy for MPS IIIB will require the development of a murine model expressing a misfolded NAGLU variant, enabling in vivo assessment of brain bioavailability and therapeutic rescue.

## 4. Conclusions

In conclusion, this study highlights the potential of glycomimetics as pharmacological chaperones for MPS IIIB. The compounds evaluated showed a variable, but significant ability to stabilize the mutated enzyme and restore its activity, with particular interest in MK 8719, whose efficacy across multiple genotypes and previously documented brain penetration make it a prime candidate for future therapeutic applications. Although further investigation is needed, particularly in iPSC-derived neuronal models and animal models expressing foldable NAGLU mutations, these results support a genotype-based therapeutic strategy and highlight glycomimetics as a promising avenue for the development of a targeted treatment for MPS IIIB.

## Figures and Tables

**Table 1:** Phenotype and genotype of SF patients.

Patient ID	Gender	Genotype (DNA)	Genotype (Protein)	Age at Sampling	Source
SF1	Unknown	c.531 + 1G > C	Likely truncated or absent protein	Unknown	CHU Lyon BioBank
SF2	Unknown	c.1444C > T	p.Arg482Trp	Unknown	
SF3	M	c.1876C > T	p.Arg626Ter	7 years	Coriell institute for medical research
SF4	M	2045T > G 419A > G	Leu682Arg Tyr140Cys	7 years	
SF5	F	457G > A	Glu153Lys	1 year	
SF6	F	889C > T	Arg297Ter Arg643His	7 years	
SF7	F	1073C > T	Pro358Leu	3 years	

**Table 2:** Summary of the maximum fold increase in NAGLU enzyme activity results observed in the different patient-derived cell lines (NE: no enhancement).

	3 (AzeNAc)	4 (DNJNAc)	5 (Thiamet G)	6 (MK 8719)	7 (α-HNJ)
SF2	72 ± 8 (at 100 µM)	55 ± 17 (at 100 µM)	46 ± 7 (at 100 µM)	117 ± 23 (at 100 µM)	NE
SF4	59 ± 11 (at 100 µM)	52 ± 9 (at 30 µM)	113 ± 10 (at 100 µM)	54 ± 10 (at 100 µM)	NE
SF5	NE	96 ± 21 (at 100 µM)	45 ± 5 (at 100 µM)	74 ± 14 (at 100 µM)	NE
SF7	107 ± 7 (at 100 µM)	85 ± 10 (at 100 µM)	4 ± 1 (at 100 µM)	65 ± 9 (at 100 µM)	NE

## Data Availability

The original contributions presented in this study are included in the article/Supplementary Materials. Further inquiries can be directed to the corresponding authors.

## Supplementary Materials

The following supporting information can be downloaded at https://www.mdpi.com/article/10.3390/biom16020313/s1, Figure S1: Molecular docking of NAGLU (PDB 4XWH) with heparan sulfate (yellow) and glycomimetics AzeNAc (3), DNJNAc (4), Thiamet G (5), MK 8719 (6), and α-homonojirimycin (α-HNJ,7), Figure S2: WST-1 cell viability assay.

## Abbreviations

The following abbreviations are used in this manuscript:

CNS	Central nervous system
DMB	1.9-dimethylmethylene blue
ERT	Enzyme replacement therapy
GAG	Glycosaminoglycan
GlcNAc	*N*-acetyl-glucosamine
GT	Gene therapy
HS	Heparan sulfate
HSCT	Hematopoietic stem cell transplant
LSDs	Lysosomal storage disorders
MPS	Mucopolysaccharidosis
NAGLU	*N*-acetyl-alpha-glucosaminidase
NT	Non-treated
OGA	*O*-linked *N*-acetylglucosaminidase; *O*-GlcNAcase
PCT	Pharmacological chaperone therapy
T_m_	Melting temperature
