# Supplementary material for: Evaluation of GlcNAc-Configured Glycomimetics as Pharmacological Chaperones of NAGLU for the Treatment of Mucopolysaccharidosis IIIB
Source: Biomolecules. 2026 Feb 16;16(2):313. doi: 10.3390/biom16020313 (PMC12938346; doi:10.3390/biom16020313)
Supplement: Supplementary file 1 [file biomolecules-16-00313-s001.zip › biomolecules-4083864-supplementary.pdf]

Supplementary information

Evaluation of GlcNAc-configured Glycomimetics as Pharmacological Chaperones of NAGLU for the Treatment of Muco-polysaccharidosis IIIB

Nissrine Ballout[1,2], Jérôme Désiré[3], Angela Johana Espejo Mojica[4,5], Katherin Niño-Traslaviña[4], Daniel Sandoval-Trujillo[5], Carlos Javier Alméciga Díaz[4,5], Yves Blériot[3*], Jérôme Ausseil[1,2*]

1 Toulouse Institute for Infectious and Inflammatory Diseases (Infinity), University of Toulouse, Inserm U1291, CNRS U5051, 31024 Toulouse, France

2 Biochemistry Laboratory, Federative Institute of Biology, CHU Toulouse, 31024 Toulouse, France

3 Organic Synthesis Team, Glycochemistry Group, IC2MP UMR CNRS 7285, Université of Poitiers, 86073 Poitiers Cedex 9, France

4 Institute for the Study of Inborn Errors of Metabolism, Faculty of Science Pontificia Universidad Javeriana Cra. 7 No. 43-82 Building 54, Lab 305A, Bogotá 110231, Colombia; cjalmeciga@javeriana.edu.co (C.J.A.-D.)

5 Dogma Biotech, Bogotá 110231, Colombia

* Correspondence: yves.bleriot@univ-poitiers.fr (Y.B.); jerome.ausseil@inserm.fr (J.A.)

Content

Supplementary Figure S1. Molecular docking of NAGLU (PDB 4XWH) with heparan sulfate (yellow) and AzeNAc (3), DNJNAc (4), Thiamet G (5), MK 8719 (6), and α-homonojirimycin (α-HNJ,7) ………………………………………… 3 glycomimetics

Supplementary Figure S2. WST-1 cell viability assay. Cells were treated with either the culture medium (NT), or the tested molecule at 10, 30, and 100μM for 48 and 72 hours (h)……………………………………………………………………………………4

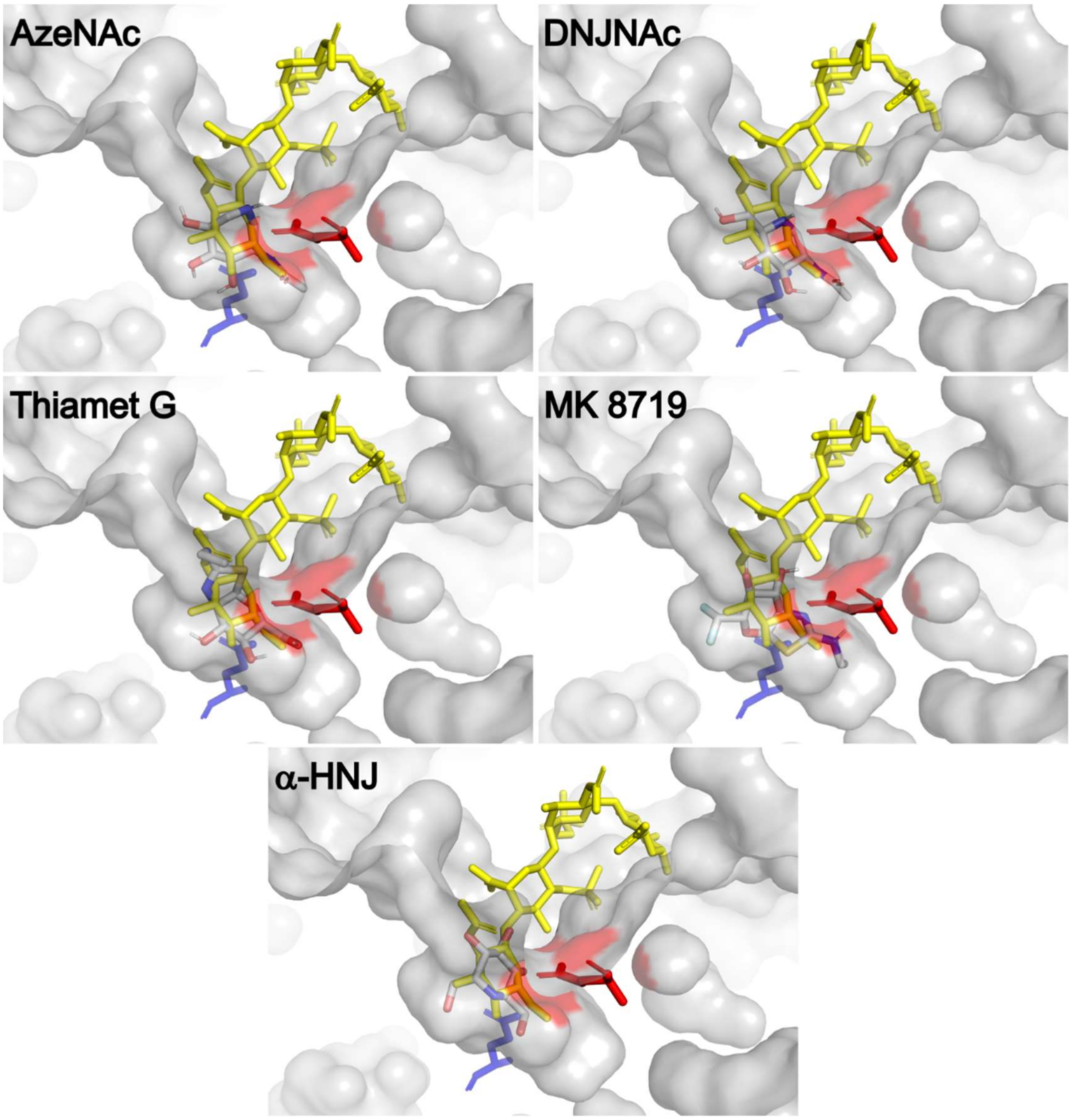

Supplementary Figure S1. Molecular docking of NAGLU (PDB 4XWH) with heparan sulfate (yellow) and glycomimetics AzeNAc (3), DNJNAc (4), Thiamet G (5), MK 8719 (6), and α-homonojirimycin (α-HNJ, 7). Catalytic residues Glu316 and Glu446, within NAG-LU active cavity, are colored in red and blue, respectively.

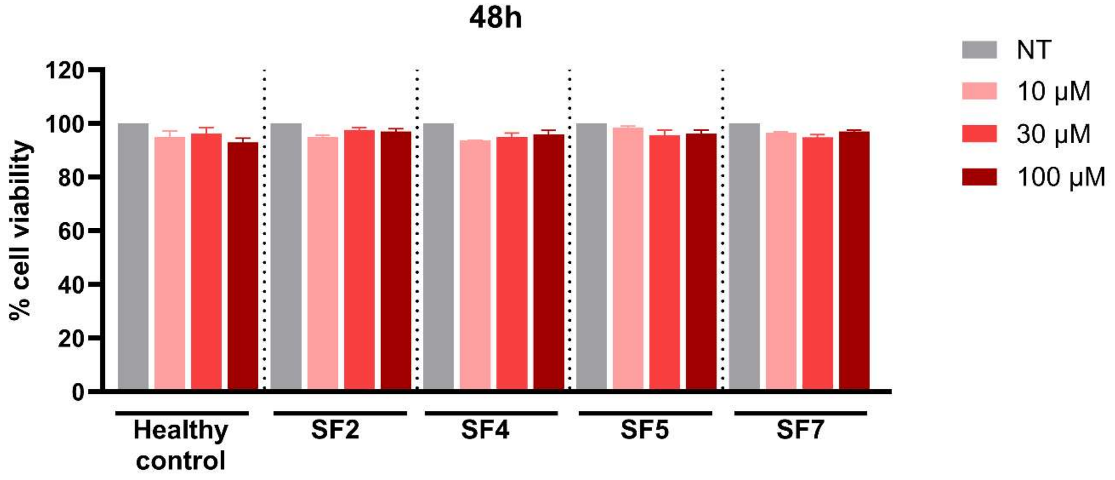

Supplementary Figure S2. WST-1 cell viability assay. Cells were treated with either the culture medium (NT), or the tested molecule at 10, 30, and 100µM for 48 and 72 hours (h).
